# Unintentional retinal artery amputation during macular peeling

**DOI:** 10.3205/oc000140

**Published:** 2020-03-18

**Authors:** Yoshiaki Shimada

**Affiliations:** 1 Department of Ophthalmology, Fujita Health University Bantane Hospital, Aichi, Japan

**Keywords:** amputation, avulsion, macular peeling, retinal artery, vitrectomy

## Abstract

**Objective:** To report a case of unintentional retina artery amputation during macular peeling.

**Methods:** Observational case report

**Results:** A 73-year-old female underwent surgery to treat epiretinal membrane (ERM) associated with severe staphyloma in her left eye. While the ERM/posterior hyaloid membrane visually enhanced with triamcinolone (TA) was peeled, a movement of the forceps unintentionally involved the inferior temporal branch artery near the inner edge of the staphyloma. The artery was avulsed and amputated. Oozing from the retinal cleft that had once enfolded the artery and microscopic bleeding from the amputation stump were observed. The vitreous hemorrhage obscured a view of the fundus more than 4 weeks after the surgery. After 8 weeks, postoperative visual acuity was improved; however, the superior nasal visual field was lost, and the patient was aware of the broken vessel as a floater in her vision.

**Conclusions:** Macular peeling is technically challenging, so meticulous attention must be paid to avoid any damage on vessels. The retina tissue was stretched in a staphyloma and vessels were bulged into the vitreous space especially at the inner edge of the staphyloma. High levels of TA dye here buried the texture of the retina. Excessive TA should be removed prior to macular peeling.

## Introduction

Macular peeling generally refers to the surgical technique for the correction of a hole or epiretinal membrane (ERM) in the macula [1]. There may be complications with this technique, such as postoperative endophthalmitis, which are common to other ocular surgeries. However, mechanical traumas directly attributable to macular peeling have also been reported [1], [2], [3], [4], [5], [6], [7], for example, punctate chorioretinopathy [2], optic nerve fiber layer dissociation [3], [4], [5], and inner retinal defects [6]. Retinal vessels may also be damaged. Garrity et al. for example reported retinal artery wall rupture [7]. Here I report a case of unintentional retinal artery amputation during macular peeling. The amputated and avulsed retinal artery was curled up into the vitreous space causing myodesopsia and a visual field defect.

## Case description

**Figure 1:**
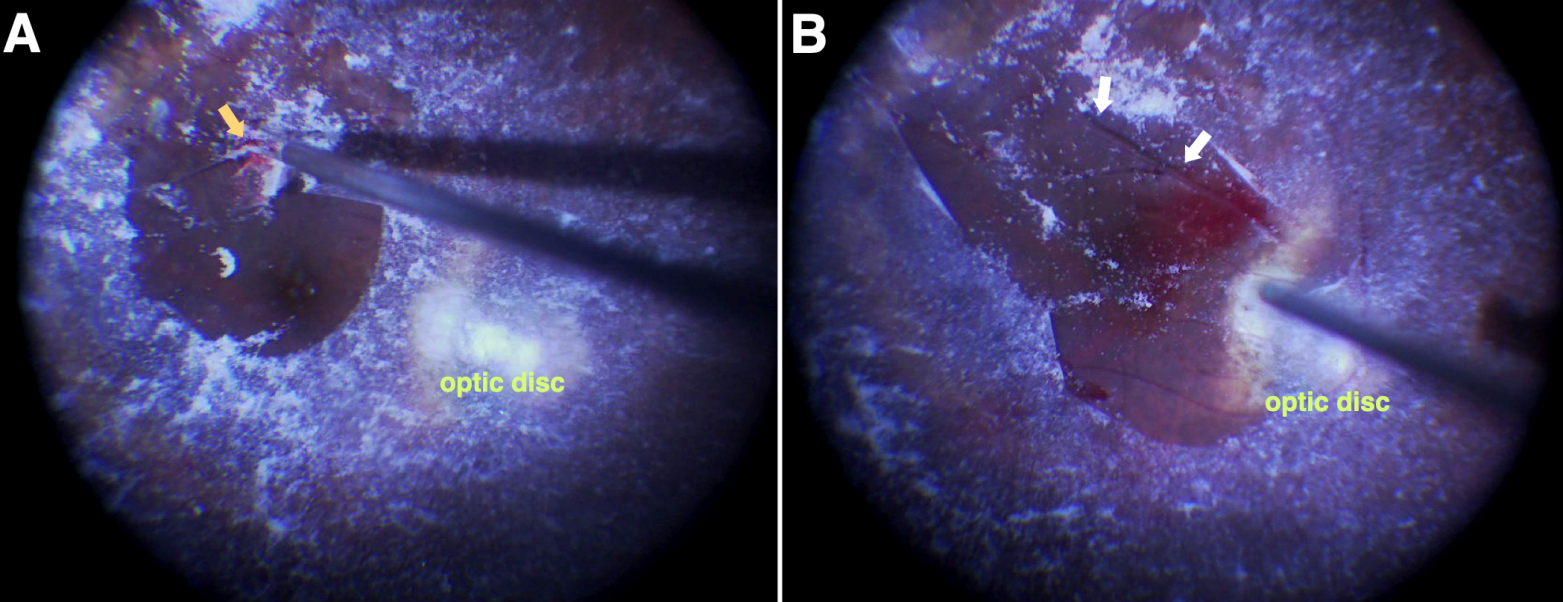
A) When the forceps picked up the epiretinal tissue, the inferior temporal retinal artery was accidentally involved (yellow arrow) and broken off. B) The broken artery (white arrows) was floating and pulsating in the vitreous space. Microscopic bleeding at the broken end and oozing from the retinal cleft that had once enfolded the artery were observed.

**Figure 2:**
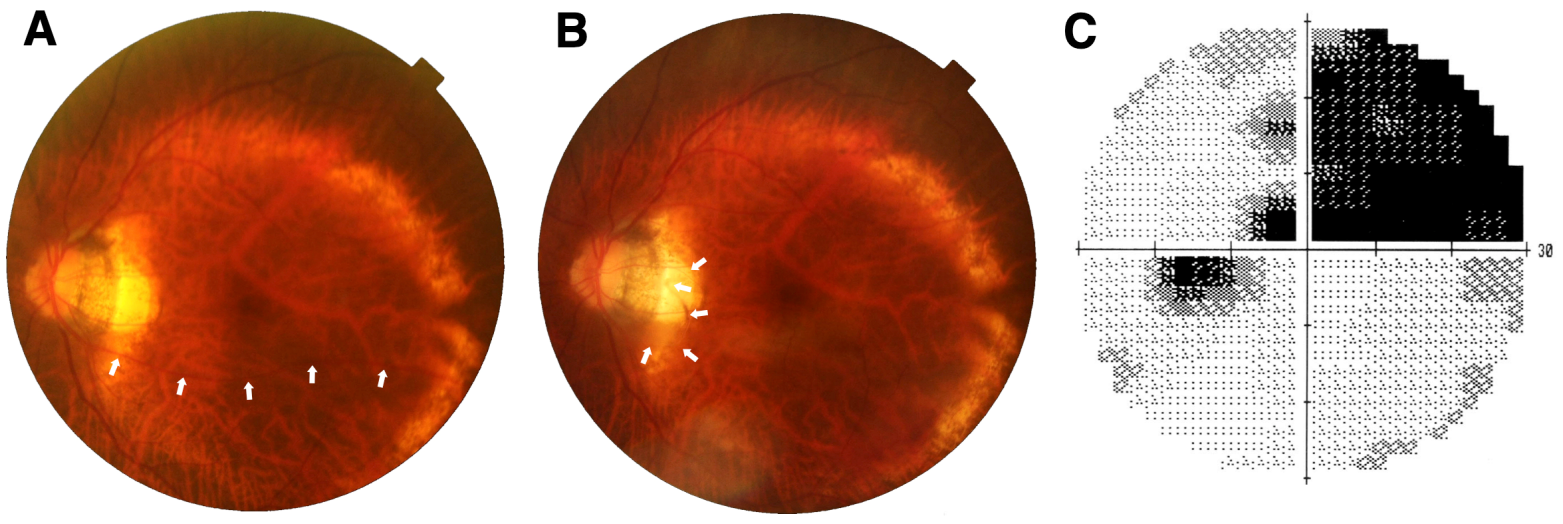
A) Preoperative: Path of the inferior temporal artery is indicated by white arrows. B) Postoperative: The artery (white arrows) is broken and curled up above the optic disc. C) Pattern deviation of visual field perimeter (Humphrey Field Analyzer, 30-2 program; Carl Zeiss Meditec, Inc., Dublin, CA, USA) obtained postoperatively from the patient’s left eye. The superior nasal field was lost.

A 73-year-old female underwent surgery to treat cataract and ERM associated with severe staphyloma in her left eye. Preoperative visual acuity was 20/40 in the left eye with –5.00 spheres and –1.50×60 degree cylinders. Phacoemulsification, pars plana vitrectomy, and intraocular lens implantation were performed. While the ERM/posterior hyaloid membrane visually enhanced with triamcinolone (TA) was peeled, a movement of the forceps unintentionally involved the inferior temporal branch artery near the inner edge of the staphyloma (Figure 1A (Fig. 1)). The artery was immediately avulsed and amputated, and began pulsating and floating in the vitreous space (Figure 1B (Fig. 1)). Oozing from the retinal canal in which the artery had been located and microscopic bleeding from the amputation stump were observed. They remained untouched while a full fluid-air exchange was performed. The vitreous hemorrhage obscured a view of the fundus more than 4 weeks after the surgery. After 8 weeks, postoperative visual acuity was improved to 20/25. The inferior temporal artery in the preoperative fundus image (Figure 2A (Fig. 2)) was curled up into the vitreous space postoperatively (Figure 2B (Fig. 2)). The patient was aware of the vessel as a floater in her vision. The superior nasal visual field was lost (Figure 2C (Fig. 2)).

## Discussion

Macular peeling is technically challenging [1], [7], [8], so meticulous attention must be paid to avoid any damage on vessels. Even if it is not amputation, the accidental breakage of an artery can cause a permanent visual field loss.

The major complications described here developed during this procedure for various reasons. First, the retina tissue was stretched in a staphyloma and vessels were bulged into the vitreous space especially at the inner edge of the staphyloma. Macular peeling can easily damage a vessel. Second, while staining with adjuvant dyes can make the procedure quicker, easier, and more effective to perform, as well as reducing mechanical trauma to the retina [1], high levels of TA dye here buried the texture of the retina. Excessive TA should be removed prior to macular peeling.

Only in cases where a retinal artery has been amputated and avulsed, cauterization to trim the floating artery close to the optic disc should be considered. This may reduce the bleeding, helping both to improve recovery and prevent the floating artery from becoming a floater in the patient’s field of vision.

## Conclusions

Unintentional retinal artery amputation during macular peeling caused myodesopsia and a visual field defect after the vitreous hemorrhage resolved.

## Notes

### Competing interests

The author declares that he has no competing interests.
